# Successful treatment of acute respiratory distress syndrome caused by hypervirulent *Klebsiella pneumoniae* with extracorporeal membrane oxygenation and continuous renal replacement therapy: A case report and literature review

**DOI:** 10.3389/fmed.2022.936927

**Published:** 2022-08-24

**Authors:** Wenzhong Peng, Yanhao Wu, Rongli Lu, Yunpeng Zheng, Jie Chen, Pinhua Pan

**Affiliations:** ^1^Department of Respiratory Medicine, National Key Clinical Specialty, Branch of National Clinical Research Center for Respiratory Disease, Xiangya Hospital, Central South University, Changsha, China; ^2^Center of Respiratory Medicine, Xiangya Hospital, Central South University, Changsha, China; ^3^Clinical Research Center for Respiratory Diseases in Hunan Province, Xiangya Hospital, Central South University, Changsha, China; ^4^Hunan Engineering Research Center for Intelligent Diagnosis and Treatment of Respiratory Disease, Xiangya Hospital, Central South University, Changsha, China; ^5^National Clinical Research Center for Geriatric Disorders, Xiangya Hospital, Central South University, Changsha, China

**Keywords:** extracorporeal membrane oxygenation (ECMO), continuous renal replacement therapy (CRRT), hypervirulent *Klebsiella pneumoniae* (hvKP), acute respiratory distress syndrome (ARDS), septic shock

## Abstract

Hypervirulent *Klebsiella pneumoniae* (hvKP) causes invasive infections and leads to high morbidity and mortality rates. Here, we report the case of a Chinese man with diabetes mellitus who developed acute respiratory distress syndrome and septic shock due to hvKP belonging to the K1 strain. The patient was treated with venovenous extracorporeal membrane oxygenation and continuous renal replacement therapy, in combination with antibiotics and recovered well. Clinicians should be aware of fatal infections caused by hvKP and investigate the best treatment options for patients at various stages of infection.

## Background

The emergence of hypervirulent *Klebsiella pneumoniae* (hvKP) poses a significant challenge to public health (1). Additionally, hvKP can cause fatal systemic infections. In contrast to classical KP, hvKP displays hypervirulent phenotypic and genotypic characteristics, namely, a hypermucoviscous phenotype and the presence of different virulence genes (2).

Hypervirulent *K. pneumoniae* can cause community-acquired or nosocomial infections in both relatively healthy individuals (3) and those with underlying diseases such as diabetes mellitus (4). It may result in septic shock and multi-organ failure, which may be life-threatening.

Here, we report the case of a patient infected with hvKP belonging to serotype K1 and with virulence-associated genes *iutA* and *rmpA*. He developed septic shock, acute respiratory distress syndrome (ARDS), and acute renal failure within a short time, and was successfully treated with veno-venous extracorporeal membrane oxygenation (vv-ECMO) and continuous renal replacement therapy (CRRT) in combination with antibiotics.

## Case presentation

The patient was a 52-year-old man who was admitted to the emergency room because of fever for 4 days and chest pain for 1 day. The patient had type 2 diabetes mellitus. He developed dyspnea with reduced oxygen saturation, and his blood pressure also declined the next morning; therefore, he was admitted to the respiratory intensive care unit (RICU). His temperature was 36.6°C, pulse rate 128 beats/min, respiratory rate 26 times/min, and blood pressure 113/62 mmHg (with a norepinephrine dose of 0.05 μg/kg/min). Coarse crackles were heard in both lungs. Endophthalmitis in the left eye was verified during ophthalmological consultation. Laboratory examinations revealed the following: white blood cell count, 1.7 × 10^9^/L; neutrophil counts 1.6 × 10^9^/L; lymphocyte counts, 0.1 × 10^9^/L; platelets, 72 × 10^9^/L; procalcitonin, 59.5 ng/ml; IL-6 > 1,000 pg/ml; TNF-α, 22.5 pg/ml; IL-1β, 9.31 pg/ml; IL-10, 37.9 pg/ml; and C-reactive protein, 470 mg/L. *K. pneumoniae* with a hypermucoviscous phenotype was isolated from the blood and bronchoalveolar lavage fluid (BALF) cultures. PCR and metagenomic next-generation sequencing (mNGS) of BALF identified a sequence type (ST) 23 serotype K1 strain with virulence-associated genes *iutA* (aerobactin) and *rmpA* (regulator of mucoid phenotype), the drug resistance gene *blaSH* was also positive. CT scan of the abdomen and brain revealed no abscess. Tracheal intubation and mechanical ventilation were performed promptly. Oliguria, a rapid decline in renal function (serum creatinine increase >100% within 48 h), and severe acidosis (pH 7.08–7.15) occurred; hence, CRRT was initiated on the 2nd day. Prone position and recruitment maneuver were attempted to improve oxygenation, but arterial blood gas analysis indicated continuous deterioration in the P/F ratio, pH, and hypercapnia.

**Figure 1:**
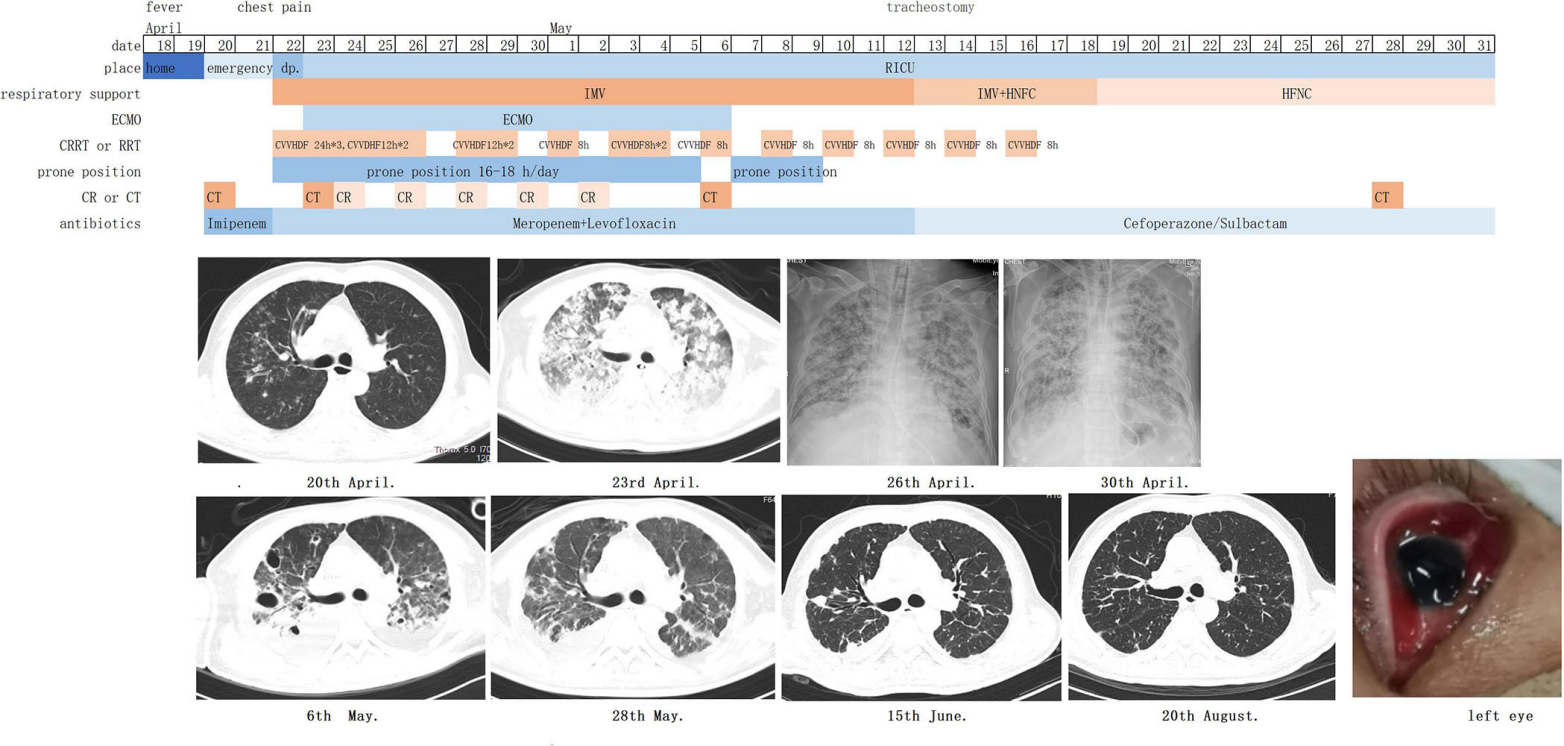
Timeline of courses and treatments. IMV, invasive mechanical ventilation; HFNC, high-flow nasal cannula oxygen therapy; ECMO, extracorporeal membrane oxygenation; CRRT, continuous renal replacement therapy; CVVHDF, continuous venovenous hemodiafiltration; CT, computerized tomography; CR, Chest radiography.

On the 3rd day after admission, vv-ECMO was performed following the assessment of cardiac function by ultrasonography, and hypoxemia and hypercapnia were corrected promptly. The prone position, CRRT, and antibiotics (meropenem and levofloxacin based on antimicrobial susceptibility test findings) were continued for the next few days. The patient's condition gradually improved, and ECMO was withdrawn successfully on the 17th day. He underwent tracheostomy on the 21st day, was supported by a ventilator and high flow nasal cannula (HFNC) in turn, and subsequently moved to full HFNC support on the 29th day. He was transferred out of the RICU on the 41st day (Table 1; Figure 1). In the follow-up, his lung (Figure 1) and renal functions were gradually restored, and his serum creatinine level dropped to 143 μmol/L on 26 April 2022, but the vision of the left eye was lost.

**Table 1:** ECMO, ventilator parameters, inflammatory markers, and renal function of the patient.

	**D1**	**D2**	**D3**	**D4**	**D5**	**D6**	**D8**	**D10**	**D13**	**D17**	**D31**
FiO_2_	0.7	0.75	0.9	0.3	0.3	0.3	0.3	0.45	0.25	0.55	0.32
PEEP (mmH_2_O)	12	12	12	15	15	12	10	10	10	10	HFNC
Plat pressure (mmH_2_O)	15	15	–	14	–	15	–	12	–	14	–
CL	26.6	–	–	15.5	–	16.1	–	27.8	–	32.5	–
Vt (ml)	399	–	–	217	–	242	–	334	–	453	–
PaO_2_ (mmHg)	63	63	61	91	89	120	86	85	82	97	123
PaCO_2_ (mmHg)	73	55	74	45	44	40	32	43	36	45	42
pH	7.09	7.15	7.36	7.49	7.45	7.42	7.39	7.41	7.43	7.35	7.48
P/F ratio	90	84	67		–	–	–	–	–	176	384
ECMO flux (L/min)	–	–	–	4.55	4.52	4.29	4.35	4.54	4.44	–	–
ECMO FiO_2_	–	–	–	1	1	1	1	1	1	–	–
NE dosage (μg/kg/min)	0.08	0.1	0.1	0.15	0.1	0.05	0.01	0	0	0	0
WBC (*10^9^)	1.7	2.0	14.8	15.8	16.4	13.9	15.4	14.2	6.4	7.6	12.5
Platelet (*10^9^)	72	41	20	24	44	42	23	17	84	141	212
N (*10^9^)	1.6	1.8	14.3	15.1	15.1	13.3	14.8	13.6	5.5	6.8	10.6
L (*10^9^)	0.1	0.2	0.3	0.4	0.7	0.4	0.4	0.4	0.4	0.4	0.8
PCT (ng/ml)	21.93	59.5	56.05	48.63	37.87	27.87	25.17	14.26	9.26	3.01	1.72
CRP (mg/L)	–		470	371	204	126	193	191	87.6	88.7	64.5
IL-6 (pg/ml)	–	–	>1000	–	–	–	275	–	–	–	–
TNF-alpha (pg/ml)	–	–	22.5	–	–	–	20.2	–	–	–	–
IL-10 (pg/ml)	–	–	37.9	–	–	–	16.9	–	–	–	–
IL-1β (pg/ml)	–	–	9.31	–	–	–	9.85	–	–	–	–
Cr (μmol/L)	126.3	204.8	150.3	242	294	273	164	194.1	348	307	351

FiO_2_, fraction of inspiration O_2_; PEEP, positive end-expiratory pressure; CL, Compliance of Lung; Vt, tidal volume; NE, Norepinephrine; WBC, white blood cell count; N, neutrophil; L, lymphocyte; PCT, procalcitonin; CRP, C-reactive protein; IL, interleukin; TNF-alpha, tumor necrosis factor.

## Discussion

Here, we present the case of a diabetic patient infected with hvKP in the community. The patient developed ARDS and septic shock in a short time. After 41 days of intensive care and support, he survived with good recovery of the lung and kidney functions but lost vision in his left eye.

Hypervirulent *K. pneumoniae* was defined as the hypermucoviscosity phenotype (string test showing a positive result) (5). Other features associated with hvKP include K1 or K2 serotype, overexpression of *rmpA* (regulator of mucoid phenotype), and stealth siderophore biosynthesis (5). The hvKP strain in our case belonged to serotype K1 with virulence genes *iutA* and *rmpA*. K1 and K2 strains are the dominant strains causing community-onset infections (6). K1 is more prevalent in Asian countries whereas K2 is more prevalent in Europe (7, 8). The K1 strain is associated with a higher incidence of liver abscess (8). Numerous studies have identified virulence genes of hvKP over the past few decades (6, 9). Since not all hvKP have the hypermucoviscous phenotype, some studies have defined the hvKP based on the positivity of virulence genes, namely, the combination of *peg-344, iroB, iucA, rmpA*, and *rmpA2* (6, 9). *RmpA* is a gene that regulates the synthesis of extracellular polysaccharide capsules and is responsible for hypermucoviscosity and is found in 95.1–99.4% of hvKP (6, 10). *IutA* is a gene encoding the aerobactin system, which is crucial for growth and infection (11), and is present in 85.3–90.7% of hvKP (10, 12). Concurrence of *rmpA* and *iutA* may increase the risk of developing a severe form of infection (13). In our case, we confirmed the presence of virulence genes in BALF by NGS. In the conventional method, we should obtain a positive culture of the specimen, and PCR is needed for the identification of the serotype, sequence type, drug resistance genes, and virulence genes, which is time-consuming. NGS can be used to analyze capsular serotypes and identify virulence-associated genes and drug resistance genes simultaneously (14). Deoxyribonucleic acid extracted from clinical specimens can directly be processed for NGS analysis. Therefore, metagenomics is advantageous for the identification of the strain and virulence of the hvKP in contrast to traditional cultivation (15). The use of NGS may help in the early and accurate identification of hvKP, ultimately improving patients' outcomes.

Hypervirulent *K. pneumoniae* has spread across Asian countries such as China, Japan, and South Korea, with sporadic but increasing rates reported elsewhere. We have summarized the reports on the hvKP from 1 January 2021 to 30 April 2022 in Table 2. The hvKP can cause severe community or hospital-acquired infections (49, 50). The risk factors include renal insufficiency (42, 51), diabetes (52), age ≥ 65 years (1), and chronic alcoholism (53). HvKP causes tissue invasive infection, often involving multiple sites. Septic shock and multi-organ failure are more common in patients infected with hvKP than in those infected by classical *K. pneumoniae* (cKP). The mortality rate of hvKP infection is higher in the elderly and those who are infected with carbapenem-resistant strains, but it appears to be similar in the general population between carbapenem-sensitive hvKP and cKP groups (12). In our study, the patient had type 2 diabetes without taking any hypoglycemics. He developed disseminated infections in the lungs and left eye. The patient progressed to septic shock and multi-organ failure. He also manifested a surge in inflammatory markers and prolonged thrombocytopenia.

**Table 2:** The case reports and studies of hvKP infections.

**References**	**Publication year**	**hvKP (n)**	**Community acquired**	**Carbapenem-resistant (n)**	**Age**	**ARDS/Severe pneumonia**	**Septic shock**	**Bacteremia**	**Intracranial infection**	**Liver abscess**	**Mortality**
Piazza et al. (16)	2022	1	1	0	75	0	0	0	1	0	0
Konagaya et al. (17)	2022	1	1	0	69	1	0	1	1	1	0
Kong et al. (18)	2022	1	1	0	24	1	1	1	0	1	0
Liu et al. (19)	2022	1	1	0	>60	0	0	1	0	1	0
Salawati et al. (20)	2021	1	1	0	58	1	1	1	0	1	1
Lin et al. (21)	2021	1	0	0	29	1	0	1	0	1	0
Kim et al. (22)	2021	1	1	0	50	0	0	1	0	1	0
Xie et al. (23)	2021	1	1	0	56	1	0	1	0	1	0
Kamau et al. (24)	2021	1	1	0	30	0	0	0	1	0	0
Marinakis et al. (25)	2021	3	3	0	51	–	–	2	3	2	3
Oh et al. (26)	2021	1	1	0	57	–	1	1	1	1	1
McHardy et al. (27)	2021	1	1	0	53	0	1	1	0	0	0
Lee et al. (28)	2021	1	1	0	63	0	0	1	0	0	0
Nakamura et al. (29)	2021	2	2	0	45, 29	0	0	2	1	1	0
JIn et al. (30)	2021	1	1	1	68	0	0	1	0	0	0
Macleod et al. (31)	2021	1	1	0	60	1	1	1	1	1	1
Horuguchi et al. (32)	2021	1	1	0	67	0	0	1	1	1	0
Hassanin et al. (33)	2021	1	1	0	55	0	0	1	0	0	0
Troché et al. (34)	2021	1	1	0	54	0	0	1	0	0	0
Chen et al. (35)	2021	1	1	0	57	1	1	1	0	0	0
Zhao et al. (36)	2021	1	0	1	68	1	1	–	–	–	1
Lan et al. (11)	2021	1	1	0	77	–	–	1	–	–	0
Khan et al. (37)	2021	1	1	0	23	0	0	1	0	0	0
Zhao et al. (38)	2021	1	1	0	80	0	0	1	0	1	0
Hosoda et al. (39)	2021	1	1	0	87	1	0	0	0	0	1
Kubota et al. (40)	2021	1	1	0	58	1	1	1	0	1	0
Himeno et al. (41)	2022	4	4	0	53 (46–72)	0	1	4	0	0	0
Anantharajah et al. (8)	2022	22	22	0	61	1	–	10	1	9	6 (27.3%)
Wei et al. (1)	2022	51	0	51	≥65	–	–	–	–	4	21 (41.2%)
Chen et al. (42)	2022	114	86	–	63 (55–73)	–	19	–	–	–	16 (14%)
Lei et al. (43)	2022	7	0	7	>60	–	–	–	–	–	5 (71.4%)
Falcone et al. (44)	2022	29	0	0	73 (65–77)	–	8	15	0	0	14 (48.3%)
Rollin et al. (45)	2021	10	10	0	58 (46–64)	–	5	–	10	–	7 (70%)
Zhou et al. (46)	2021	16	0	16	83 ± 10	–	–	–	–	–	9 (56.2%)
Li et al. (47)	2021	9	0	9	50 ± 13	–	–	–	–	9	5 (55.6%)
Kong et al. (48)	2021	6	0	6	29–81	–	–	–	–	–	3 (50%)

“–” means no information in the reports.

Veno-venous extracorporeal membrane oxygenation is increasingly used in patients with severe ARDS to correct life-threatening hypoxemia and serves as a bridge for recovery. However, vv-ECMO may not always be appropriate in patients with ARDS and septic shock. In the Piotr Suwalski study (54), 7.1% of patients on vv-ECMO due to ARDS caused by COVID-19 were converted to venoarterial (va)-ECMO when septic shock developed. Han (55) reported 23 patients with refractory septic shock treated with va-ECMO, and five patients survived. Falk et al. (56) showed that patients with septic shock with or without left ventricular failure benefited from va-ECMO more than vv-ECMO, but in their study, 6 of the 10 patients with vv-ECMO survived, possibly because vv-ECMO could improve heart function by increasing cardiac oxygenation. In our case, the patient experienced ARDS and septic shock due to hvKP. His heart function was assessed using echocardiography, and the results showed no left or right ventricular dysfunction. His norepinephrine dose was <0.2 μg/kg/min. Therefore, we decided to establish vv-ECMO, and the patient improved and avoided transitioning to V-A or hybrid ECMO. Patients with refractory septic shock (high dose of vasopressin, and inability to sustain mean arterial pressure ≥65 mmHg) should be treated with va-ECMO; however, for those with a low dose of vasopressin and no heart dysfunction, vv-ECMO may also be adequate to improve the patients' outcome.

Approximately 41.4–64.4% of critically ill patients with severe sepsis or septic shock develop acute kidney injury (57, 58). Renal replacement therapy (RRT) is widely used for these patients. However, the optimal RRT modality remains controversial. A recent meta-analysis showed no significant difference in patients and kidney survival among patients receiving CRRT, sustained low-efficiency dialysis, or intermittent hemodialysis (59). Studies on early vs. late initiation of RRT revealed no benefit for patients who start early RRT (60, 61). However, for patients with complications such as obvious fluid overload, acute pulmonary edema, severe acidosis, and severe hyperkalemia, RRT may be performed urgently. Regarding CRRT, investigators had to provide treatment continuously for 24 h with a change of membranes at least every 72 h, and a minimum ultrafiltration (or dialysate) rate of 25 ml/kg/h was recommended (61, 62). For intermittent hemodialysis, the length of the session had to be 4–6 h or more, and the frequency had to be at least once every 48 h. Recommendations were to set a blood flow rate of 150–250 ml/min, and a dialysate flow rate of 300–500 ml/min (61, 62). Across the studies, RRT dependence among survivors was about 5.9–23.1% in 28 days, lowering to 2–13.4% in 90 days (61–63). In our case, the patient developed oliguria, pulmonary edema, and severe acidosis on the 1st day after admission to the RICU. The average fluid intake of the patient was >2,500 ml/day in the first week, and early initiation of CRRT could control the fluid disturbances and prevent the deterioration of lung oxygenation and heart function. We treated the patient with continuous techniques in the first 72 h and changed to intermittent hemodialysis for 8–12 h each time on the next days (Figure 1). The patient's kidney functions gradually recovered and RRT was discontinued on the 25th day. Like COVID-19 infection, inflammatory mediators play an important role in sepsis and septic shock, exacerbating organ damage and correlating with disease severity (64). Many clinical strategies have been attempted to reduce inflammatory damage. CRRT with a specific filter may successfully lower the levels of cytokines such as TNF-α, IL-6, IL-8, and IFNγ (65). In our case, hvKP led to a severe cytokine storm with IL-6 > 1,000 pg/ml, and an extended duration of continuous venovenous hemodiafiltration seemed to remove the inflammatory mediators and restore the homeostasis of the patient. Whether the combination of CRRT and ECMO can improve the outcome of patients with septic shock and ARDS, and when and which modality should be initiated requires further study.

## Conclusion

This is a successful case of treating severe infection and multi-organ failure caused by hvKP, demonstrating that vv-ECMO and CRRT may improve the outcomes in this group of patients.

## Data availability statement

The raw data supporting the conclusions of this article will be made available by the authors, without undue reservation.

## Ethics statement

The studies involving human participants were reviewed and approved by Institutional Review Boards (IRBs) in Xiangya Hospital, Central South University. The patients/participants provided their written informed consent to participate in this study. Written informed consent was obtained from the individual(s) for the publication of any potentially identifiable images or data included in this article.

## Author contributions

WP and YW reviewed the lectures and wrote the manuscript. RL and YZ collected and arranged the materials. JC and PP viewed the complete manuscript. All authors contributed to the article and approved the submitted version.

## Funding

This study was supported by National Key R&D Program of China (No. 2016YFC1304204); Key Program of Hunan Province (No. 2022SK2038); the Project Program of National Clinical Research Center for Geriatric Disorders (Xiangya Hospital, Grant No. 2020LNJJ05).

## Conflict of interest

The authors declare that the research was conducted in the absence of any commercial or financial relationships that could be construed as a potential conflict of interest.

## Publisher's note

All claims expressed in this article are solely those of the authors and do not necessarily represent those of their affiliated organizations, or those of the publisher, the editors and the reviewers. Any product that may be evaluated in this article, or claim that may be made by its manufacturer, is not guaranteed or endorsed by the publisher.
